# Lower pretreatment HBV DNA levels are associated with better off-treatment outcomes after nucleo(s)tide analogue withdrawal in patients with HBeAg-negative chronic hepatitis B: A multicentre cohort study

**DOI:** 10.1016/j.jhepr.2023.100790

**Published:** 2023-05-12

**Authors:** Milan J. Sonneveld, Shao-Ming Chiu, Jun Yong Park, Sylvia M. Brakenhoff, Apichat Kaewdech, Wai-Kay Seto, Yasuhito Tanaka, Ivana Carey, Margarita Papatheodoridi, Piero Colombatto, Florian van Bömmel, Thomas Berg, Fabien Zoulim, Sang Hoon Ahn, George N. Dalekos, Nicole S. Erler, Maurizia Brunetto, Heiner Wedemeyer, Markus Cornberg, Man-Fung Yuen, Kosh Agarwal, Andre Boonstra, Maria Buti, Teerha Piratvisuth, George Papatheodoridis, Chien-Hung Chen, Benjamin Maasoumy, for the CREATE study group

**Affiliations:** 1 Department of Gastroenterology and Hepatology, Erasmus MC University Medical Center, Rotterdam, the Netherlands; 2 Department of Internal Medicine, Koahsiung Chang Gung Memorial Hospital, Kaohsiung, Taiwan; 3 Department of Internal Medicine, Yonsei University College of Medicine, Seoul, South Korea; 4 Faculty of Medicine, Prince of Songkla University, Hatyai, Thailand; 5 Department of Medicine, State Key Laboratory for Liver Research, The University of Hong Kong, Hong Kong; 6 Department of Gastroenterology and Hepatology, Kumamoto University, Kumamoto, Japan; 7 Institute of Liver Studies, King’s College Hospital, London, UK; 8 Department of Gastroenterology, ‘Laiko’ General Hospital of Athens, National and Kapodistrian University of Athens, Athens, Greece; 9 Hepatology Unit, University Hospital of Pisa, Pisa, Italy; 10 Division of Hepatology, Department of Medicine II, Leipzig University Medical Center, Leipzig, Germany; 11 INSERM Unit 1052, Lyon, France; 12 Medicine and Research Laboratory of Internal Medicine, National Expertise Center of Greece in Autoimmune Liver Diseases, Full Member of the European Reference Network on Hepatological Diseases (ERN RARE-LIVER), General University Hospital of Larissa, Larissa, Greece; 13 Department of Biostatistics, Erasmus MC University Medical Center, Rotterdam, the Netherlands; 14 Department of Epidemiology, Erasmus MC University Medical Center, Rotterdam, the Netherlands; 15 Department of Gastroenterology and Hepatology, Hannover Medical School, Hannover, Germany; 16 Liver Unit, Hospital Universitari Vall d’Hebron and Ciberehd del Intituto Carlos III de Barcelona, Spain

**Keywords:** HBV DNA, HBsAg, HBcrAg, HBsAg loss, Entecavir, Tenofovir

## Abstract

**Background & Aims:**

Pretreatment predictors of finite nucleo(s)tide analogue (NUC) therapy remain elusive. We studied the association between pretreatment HBV DNA levels and outcomes after therapy cessation.

**Methods:**

Patients with chronic hepatitis B who were HBeAg negative at the start of NUC treatment were enrolled from sites in Asia and Europe. We studied the association between pretreatment HBV DNA levels and (1) clinical relapse (defined as HBV DNA >2,000 IU/ml + alanine aminotransferase >2 × the upper limit of normal or retreatment) and (2) HBsAg loss after NUC withdrawal.

**Results:**

We enrolled 757 patients, 88% Asian, 57% treated with entecavir, with a median duration of treatment of 159 (IQR 156–262) weeks. Mean pretreatment HBV DNA levels were 5.70 (SD 1.5) log IU/ml and were low (<20,000 IU/ml) in 150 (20%) and high (>20,000 IU/ml) in 607 (80%). The cumulative risk of clinical relapse at 144 weeks after therapy cessation was 22% among patients with pretreatment HBV DNA levels <20,000 IU/ml *vs*. 60% among patients with pretreatment HBV DNA levels >20,000 IU/ml, whereas the cumulative probabilities of HBsAg loss were 17.5% *vs*. 5% (*p* <0.001). In multivariable analysis, pretreatment HBV DNA levels <20,000 IU/ml were independently associated with a reduced likelihood of clinical relapse (adjusted hazard ratio 0.379, *p* <0.001) and with an increased chance of HBsAg loss (adjusted hazard ratio 2.872, *p* <0.001).

**Conclusions:**

Lower pretreatment HBV DNA levels are associated with a lower risk of clinical relapse and a higher chance of HBsAg loss after cessation of NUC therapy, independent of end-of-treatment viral antigen levels. Further studies are needed to confirm these findings in non-Asian populations.

**Impact and Implications:**

A subgroup of patients with chronic hepatitis B may not require retreatment after stopping antiviral therapy. In this study, comprising 757 patients with chronic hepatitis B from Europe and Asia, we found that higher viral load before initiation of treatment was a risk factor for relapse after stopping treatment. Patients with a low HBV DNA level before starting antiviral therapy had the lowest risk of relapse, and a high chance of HBsAg loss, after stopping treatment. These findings can help select patients for treatment withdrawal and guide intensity of off-treatment monitoring.

## Highlights

• A subset of patients may achieve sustained remission and even HBsAg loss after cessation of nucleo(s)tide analogue therapy.
• Higher pretreatment HBV DNA levels were associated with a higher risk of clinical relapse, and a lower chance of HBsAg loss, after cessation of treatment.
• A pretreatment HBV DNA level <20,000 IU/ml was associated with favourable outcomes after cessation of antiviral therapy.
• The association between pretreatment HBV DNA levels and off-treatment outcomes was independent of other established predictors, including end-of-treatment HBsAg and HBcrAg levels.

## Graphical abstract

**Figure:**
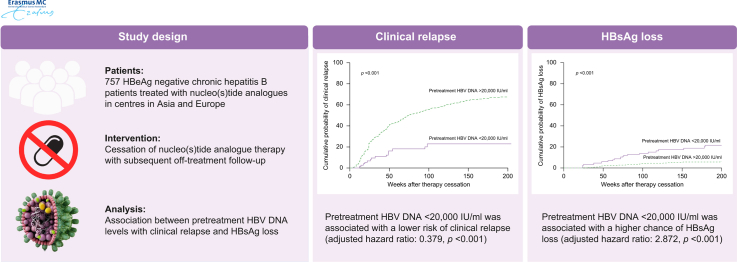

## Introduction

Long-term treatment with nucleo(s)tide analogues (NUCs) results in HBV DNA suppression in the vast majority of patients with chronic hepatitis B (CHB). Suppression of HBV DNA is associated with histological improvement and a reduced risk of hepatocellular carcinoma.^1^ Unfortunately, clearance of HBsAg (*i.e*. functional cure) is achieved in only a minority of patients despite long-term HBV DNA suppression. Interestingly, recent studies indicate that a subset of patients may achieve persistently low HBV DNA levels and even HBsAg clearance after cessation of NUC therapy. Such favourable outcomes are more likely to be achieved in non-Asian patients, and in those with low end-of-treatment (EOT) viral antigen and/or HBV RNA levels.[2], [3], [4], [5] Unfortunately, most available predictors of successful therapy withdrawal are either not modifiable (age, ethnicity, and HBV genotype) or based on values obtained at the time of treatment cessation (*e.g.* alanine aminotransferase [ALT] and viral antigen levels). These findings can therefore not be used to counsel currently untreated patients who are considering antiviral therapy regarding their chances of finite therapy. The association between low EOT viral antigen levels and low relapse rates suggests that patients with low replicative CHB are more likely to achieve successful therapy withdrawal. Indeed, previous small studies indicate that low pretreatment HBV DNA levels may also be associated with favourable outcomes after therapy cessation, although findings are conflicting.^6^^,^^7^ However, if low pretreatment viraemia is a predictor of successful therapy withdrawal, this could be of major relevance for counselling HBeAg-negative patients with relatively low HBV DNA levels in whom treatment indications remain a matter of debate, and for assessment of relapse risk among patients being considered for enrolment in trials investigating new finite treatment regimes.

The aim of the current study was therefore to assess the relationship between pretreatment HBV DNA levels and off-treatment outcomes after cessation of NUC therapy.

## Patients and methods

### Patients

The current study used data from a pooled dataset comprising patients with CHB who discontinued NUC therapy as part of studies or clinical practice in centres in Europe and Asia.^2^^,^^3^^,^^8^ Patients were eligible for the current analysis if they had been treated with only NUCs (no history of pegylated interferon add-on was allowed) and if they (1) were HBeAg negative at the time of treatment initiation, (2) had available data on HBV DNA levels before therapy initiation, (3) had undetectable HBV DNA at NUC cessation, and (4) had available data on both HBsAg and hepatitis B core-related antigen (HBcrAg) levels at treatment cessation. Based on these selection criteria, we identified 757 patients for further analysis. This research was conducted in accordance with both the Declarations of Helsinki. The individual studies were approved by local ethical committees, and patients provided written informed consent whenever applicable.

### Laboratory testing and assessment of hepatic fibrosis

HBV DNA was measured using local PCR methods. HBsAg was measured using various standardised methods. HBcrAg was quantified with the Lumipulse G HBcrAg assay (Fujirebio Europe, Ghent, Belgium) on a LUMIPULSE G1200 analyser (Fujirebio Inc., Tokyo, Japan). The assay’s lower limit of quantification is 3 log U/ml, and the lower limit of detection is 2 log U/ml. HBV genotyping was performed using various methods including line-probe assays, restriction fragment length polymorphism, and/or sequencing. Other biochemical tests were performed using local laboratory facilities. Stage of liver disease was assessed based on liver stiffness or biopsy (when available). Cirrhosis could also be ruled in based on compatible imaging findings.

### Endpoints and statistical analysis

The association between pretreatment HBV DNA levels and EOT HBcrAg and HBsAg levels was explored using Pearson correlation and multivariable linear regression adjusting for patient age and duration of antiviral therapy. Clinical relapse was defined as the occurrence of either HBV DNA >2,000 IU/ml with ALT >2 × the upper limit of normal or re-initiation of antiviral therapy (for any reason). Patients achieving HBsAg loss were considered to remain free from clinical relapse. HBsAg loss was defined as undetectable HBsAg at any time during off-treatment follow-up. Retreated patients were considered persistently HBsAg positive. Cumulative probabilities of either endpoint were estimated using the Kaplan–Meier estimator in the overall population, across HBV DNA level categories (<20,000 *vs*. >20,000 IU/ml), and stratified by EOT HBsAg (<100 and >100 IU/ml) or HBcrAg levels (undetectable and detectable) and compared using log-rank tests. We also performed multivariable Cox regression analyses, which were adjusted for previously reported predictors of outcome after therapy withdrawal, including antiviral agent, patient ethnicity (or HBV genotype), age, sex, EOT viral antigen levels, and baseline and EOT ALT levels.

Analyses were performed using SPSS version 28.0 (IBM, Armonk, NY, USA). All statistical tests were two-sided and were evaluated at the 0.05 level of significance.

## Results

### Cohort characteristics

**Fig. 1:**
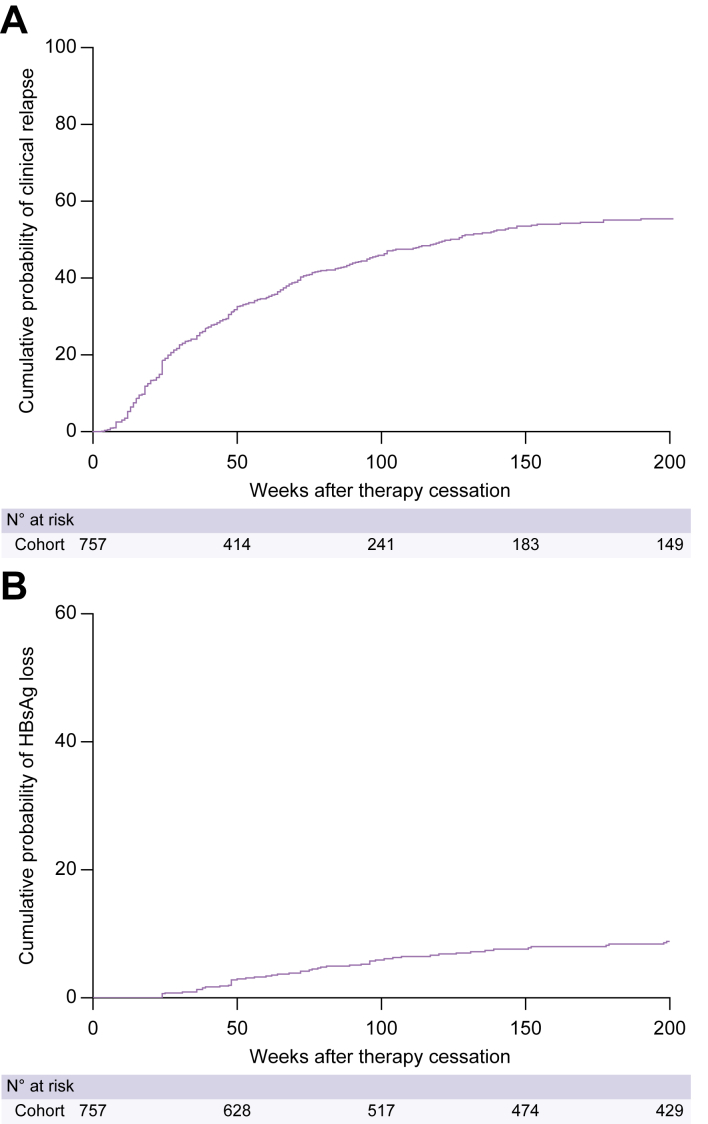
Off-treatment outcomes after cessation of antiviral therapy. The cumulative probability of (A) clinical relapse and (B) HBsAg loss.

We enrolled 757 patients, the majority of whom were treated with entecavir (56.5%; Table 1). The median duration of antiviral therapy was 159 (IQR 156–262) weeks, 157 (IQR 156–209) weeks for Asians and 368 (IQR 260–477) weeks for non-Asians (*p* <0.001). Information on the presence of cirrhosis was unavailable for 309 patients; among the remaining 448 patients, 23 (5.1%) had cirrhosis. Cohort characteristics are shown according to pretreatment HBV DNA level in Table S1 and according to ethnicity in Table S2. The cumulative probability of clinical relapse was 31% at 48 weeks, 46% at 96 weeks, and 54% at 144 weeks (Fig. 1A). The cumulative probability of HBsAg loss was 1.4% at 48 weeks, 4.1% at 96 weeks, and 6% at 144 weeks (Fig. 1B).

**Table 1:** **Cohort characteristics**.

Characteristics	N = 757
Demography	
Age (years), median (IQR)	53 (45–60)
Male, n (%)	565 (74.6)
Duration of therapy (weeks), median (IQR)	159 (156–262)
Pretreatment ALT (median, IQR)	115 (74–232)
Cirrhosis (n = 448), n (%)	23 (5.1)
Ethnicity, n (%)	
Asian	667 (88)
Caucasian	70 (9.2)
Other	20 (2.6)
Treatment, n (%)	
ETV	428 (56.5)
TDF	238 (31.4)
Other∗	91 (12.0)
HBV genotype (n = 629), n (%)	
A	10 (1.6)
B	349 (46.1)
C	184 (24.3)
D	63 (8.3)
Other	23 (3.0)
Pretreatment HBV DNA, n (%)	
<20,000 IU/ml	150 (20)
>20,000 IU/ml	607 (80.2)
EOT HBsAg, n (%)	
<100 IU/ml	200 (26.4)
>100 IU/ml	557 (73.6)
EOT HBcrAg, n (%)	
Undetectable	237 (31.3)
Detectable	520 (68.7)

ALT, alanine aminotransferase; EOT, end of treatment; ETV, entecavir; HBcrAg, hepatitis B core-related antigen; TDF, tenofovir.

∗ Lamivudine, telbivudine, or combination therapy.

### Association between EOT viral antigen levels and pretreatment HBV DNA

Mean pretreatment HBV DNA was 5.70 (SD 1.5) log IU/ml; pretreatment HBV DNA levels were <20,000 IU/ml in 150 (20%) and >20,000 IU/ml in 607 (80%). Mean HBsAg levels at EOT were 2.46 (SD 0.9) log IU/ml, and mean HBcrAg levels at EOT were 3.11 (SD 1.0) log U/ml. We observed a positive, albeit weak, correlation between pretreatment HBV DNA and EOT HBcrAg (r = 0.280, *p* <0.001) and EOT HBsAg levels (r = 0.155, *p* <0.001). These associations were consistent in multivariable linear regression adjusted for age and duration of therapy (HBsAg, beta 0.236, *p* <0.001; HBcrAg, beta 0.441, *p* <0.001).

### Low pretreatment HBV DNA levels are associated with favourable outcomes after therapy withdrawal

**Fig. 2:**
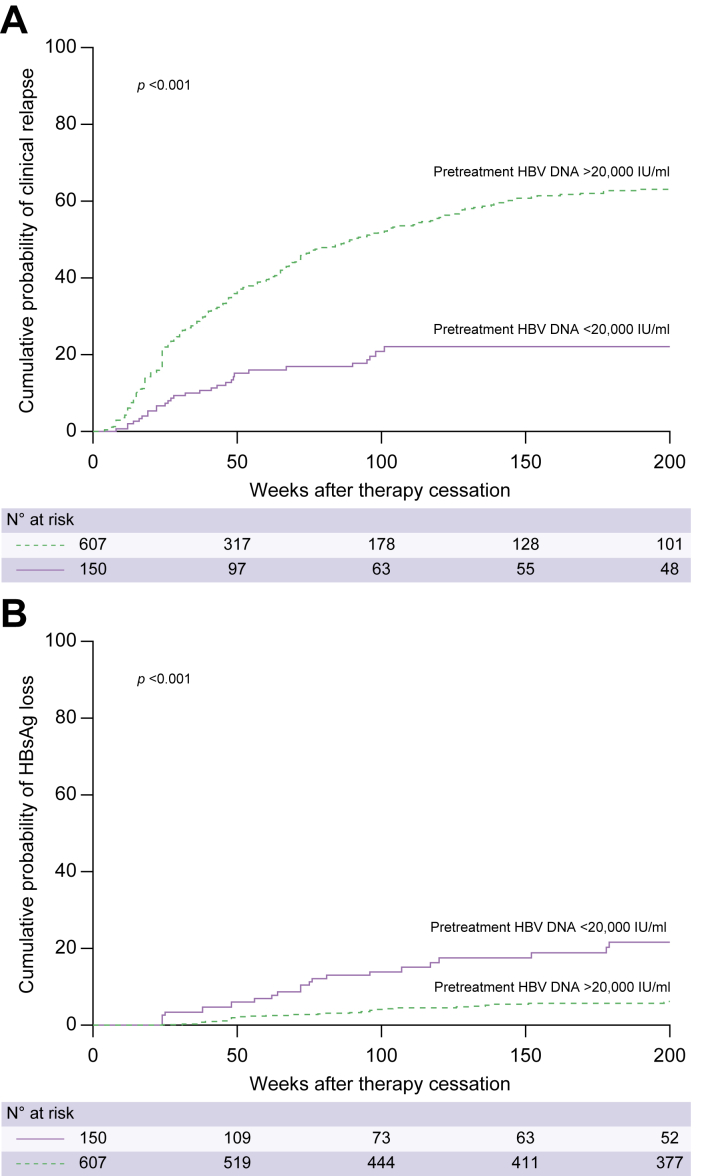
Off-treatment outcomes according to pre-treatment HBV DNA levels. Cumulative probability of (A) clinical relapse (*p* <0.001 by log-rank test) and (B) HBsAg loss (*p* <0.001 by log-rank test) according to pretreatment HBV DNA levels.

Patients with low pretreatment HBV DNA levels had significantly lower rates of clinical relapse and significantly higher chances of HBsAg clearance after therapy withdrawal (Fig. 2A and B). At 144 weeks after therapy discontinuation, the cumulative risk of relapse was 22% among patients with a pretreatment HBV DNA level <20,000 IU/ml, *vs*. 60% among patients with pretreatment HBV DNA levels >20,000 IU/ml, whereas the cumulative probability of HBsAg loss was 17.5% *vs*. 5% (*p* <0.001 for both comparisons).

### Pretreatment HBV DNA <20,000 IU/ml is associated with favourable outcomes after therapy withdrawal independent of EOT viral antigen levels

**Fig. 3:**
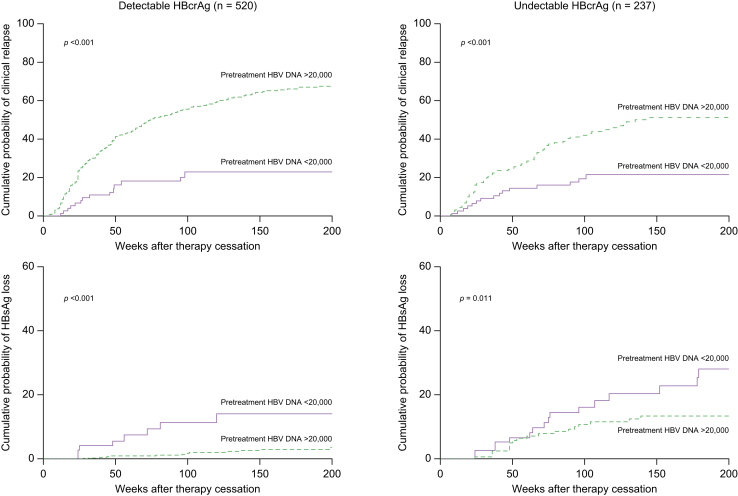
Off-treatment outcomes according to pretreatment HBV DNA levels stratified by end-of-treatment HBcrAg levels. Cumulative probability of clinical relapse and HBsAg loss according to pre-treatment HBV DNA levels for patients with detectable (n = 520; 74 [14%] with pretreatment HBV DNA <20,000 IU/ml) and undetectable HBcrAg (n = 237; 76 [32%] with pretreatment HBV DNA <20,000 IU/ml) at the end of treatment. A pretreatment HBV DNA level <20,000 IU/ml was associated with a lower cumulative risk of clinical relapse (*p* <0.001 by log-rank test) and a higher cumulative chance of HBsAg loss (*p* <0.001 by log-rank test) among patients with detectable HBcrAg at the end of treatment; similar results were obtained among patients with undetectable HBcrAg at the end of treatment (*p* <0.001 for clinical relapse and *p* = 0.011 for HBsAg loss). HBcrAg, hepatitis B core-related antigen.

**Fig. 4:**
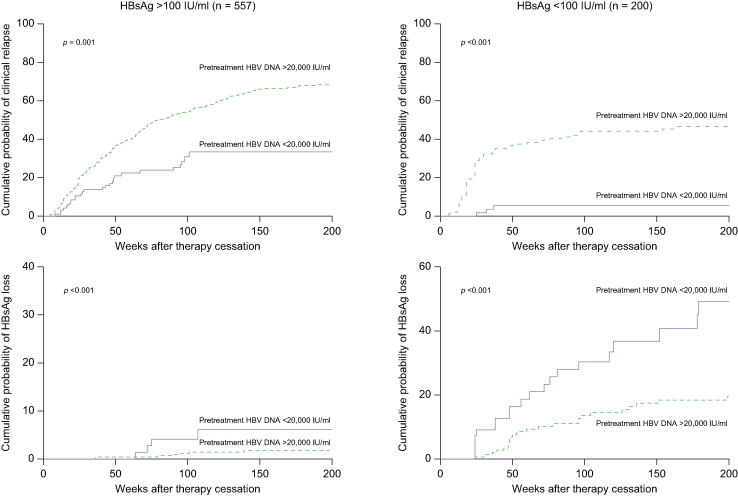
Off-treatment outcomes according to pretreatment HBV DNA levels stratified by end-of-treatment HBsAg levels. Cumulative probability of clinical relapse and HBsAg loss according to pretreatment HBV DNA levels for patients with end-of-treatment HBsAg levels above (n = 557; 95 [17.1%] with pretreatment HBV DNA <20,000 IU/ml) or below 100 IU/ml (n = 200; 55 [27.5%] with pretreatment HBV DNA <20,000 IU/ml). A pretreatment HBV DNA level <20,000 IU/ml was associated with a lower cumulative risk of clinical relapse (*p* = 0.001 by log-rank test) and a higher cumulative chance of HBsAg loss (*p* <0.001 by log-rank test) among patients with HBsAg >100 IU/ml at the end of treatment; similar results were obtained among patients with HBsAg <100 IU/ml at the end of treatment (*p* <0.001 for clinical relapse and *p* <0.001 for HBsAg loss).

Pretreatment HBV DNA levels <20,000 IU/ml were associated with a lower cumulative probability of clinical relapse and a higher cumulative probability of HBsAg loss among patients with detectable and patients with undetectable EOT HBcrAg levels (Fig. 3). Similar results were obtained in the subgroups of patients with low (<100 IU/ml) and high (>100 IU/ml) EOT HBsAg levels (Fig. 4).

### Lower pretreatment HBV DNA levels are independently associated with favourable outcomes after therapy withdrawal in multivariable Cox regression analysis

In multivariable Cox regression analysis, pretreatment HBV DNA levels <20,000 IU/ml (adjusted hazard ratio 0.379, *p* <0.001), undetectable HBcrAg, HBsAg levels <100 IU/ml, younger age, lower ALT levels, non-tenofovir therapy, and non-Asian ethnicity were associated with a reduced risk of clinical relapse after therapy cessation (Table 2). Pretreatment HBV DNA levels <20,000 IU/ml (adjusted hazard ratio 2.872, *p* <0.001), undetectable HBcrAg, HBsAg levels <100 IU/ml, and non-Asian ethnicity were also independently associated with an increased chance of HBsAg loss after treatment cessation (Table 3). Findings were consistent in a multivariable model incorporating HBV genotype instead of ethnicity (Tables S2 and S3).

**Table 2:** Factors associated with clinical relapse after nucleo(s)tide analogue withdrawal in multivariable Cox regression analysis.

Variable	HR (95% CI)	*p* value
Pretreatment HBV DNA <20,000 IU/ml	0.379 (0.262–0.550)	<0.001
EOT HBcrAg undetectable	0.635 (0.493–0.817)	<0.001
EOT HBsAg <100 IU/ml	0.569 (0.433–0.748)	<0.001
Age	1.023 (1.013–1.033)	<0.001
EOT ALT	1.004 (1.001–1.008)	0.023
TDF therapy	1.300 (1.032–1.637)	0.026
Sex	1.184 (0.921–1.521)	0.187
Asian ethnicity	1.947 (1.205–3.147)	0.006

ALT, alanine aminotransferase; EOT, end of treatment; HBcrAg, hepatitis B core-related antigen; HR, hazard ratio; TDF, tenofovir.

**Table 3:** **Factors associated with HBsAg loss after nucleo(s)tide analogue withdrawal in multivariable Cox regression analysis**.

Variable	HR (95% CI)	*p* value
Pretreatment HBV DNA <20,000 IU/ml	2.872 (1.790–4.609)	<0.001
EOT HBcrAg undetectable	2.042 (1.262–3.304)	0.004
EOT HBsAg <100 IU/ml	7.551 (4.653–12.252)	<0.001
Age	0.985 (0.963–1.007)	0.184
EOT ALT	0.989 (0.975–1.002)	0.097
TDF therapy	0.996 (0.602–1.650)	0.989
Sex	1.196 (0.711–2.014)	0.500
Asian ethnicity	0.342 (0.176–0.665)	0.002

ALT, alanine aminotransferase; EOT, end of treatment; HBcrAg, hepatitis B core-related antigen; HR, hazard ratio; TDF, tenofovir.

## Discussion

Withdrawal of NUCs is being explored as a novel approach for achieving finite therapy in CHB. The current multicentre study shows that lower pretreatment HBV DNA levels are associated with favourable outcomes after therapy cessation. These findings can be used to select currently treated patients for therapy withdrawal and to counsel currently untreated patients regarding their chances of future finite NUC therapy.

NUCs are the mainstay of treatment in most patients with CHB. NUC therapy reduces the risk of hepatocellular carcinoma and may even result in regression of liver cirrhosis in some patients.^9^ Nevertheless, long-term therapy is associated with non-compliance and in some cases relevant side effects such as renal impairment and bone mineral density decline.^10^^,^^11^ Based on encouraging results obtained in Asian and European cohorts, finite NUC therapy is being explored as a novel therapeutic approach. Although therapy withdrawal has been associated with almost universal recurrence of HBV viraemia, a substantial proportion of patients may achieve low or even undetectable HBV DNA levels through long-term off-treatment follow-up.^2^^,^^3^^,^^12^^,^^13^ Furthermore, therapy cessation may actually increase the chance of HBsAg loss, an outcome that is exceedingly rare in long-term NUC-treated patients.^3^^,^^13^ Recent studies have identified several factors associated with favourable outcomes after NUC withdrawal, which include patient age, ethnicity, fibrosis stage, HBV genotype, immune profiles, and lower EOT levels of ALT, HBsAg, HBcrAg, and HBV RNA.^2^^,^^3^^,^[14], [15], [16] Importantly, none of these factors are modifiable, and most are ascertained at the time of therapy cessation. The association between low viral antigen levels and sustained remission after therapy cessation suggests that patients with less active intrahepatic viral replication might be less likely to relapse after therapy cessation. Whether this also applies to pretreatment viraemia is unclear. Previous studies have yielded conflicting results regarding the association between pretreatment viremia and off-treatment outcomes. Among patients treated with pegylated interferon, higher pretreatment HBV DNA levels are associated with a higher likelihood of therapy failure.^17^ A recent meta-analysis of predictors of successful NUC withdrawal did not show a clear association between pretreatment HBV DNA levels and off-treatment outcomes.^6^ However, it is important to note that studies on NUC withdrawal could not always adjust for other important factors such as patient age, ethnicity, and pretreatment HBeAg status. The latter factor may be of particular relevance as HBeAg-positive patients may have both higher pretreatment HBV DNA levels and may be more likely to require retreatment.^18^ Careful analysis adjusting for these factors is therefore of major importance.

In the current multicentre study, we therefore focused specifically on patients with negative pretreatment HBeAg to avoid any influence of HBeAg status on the observed associations. In our cohort comprising 757 patients, lower pretreatment HBV DNA levels, particularly <20,000 IU/ml, were associated with both lower rates of clinical relapse and a higher chance of HBsAg clearance. These associations were consistent across patients with low and high EOT viral antigen levels and in multivariable analysis adjusting for other potential predictors of off-treatment outcomes.

Our findings are of major clinical relevance. First, our data indicate that the pretreatment HBV DNA level is a valuable new tool for selecting NUC-treated patients for therapy withdrawal. Cessation of NUC therapy in patients with pretreatment HBV DNA levels <20,000 IU/ml is associated with a low risk of relapse and is associated with a high chance of HBsAg clearance that far exceeds the rates expected with continued NUC treatment. If confirmed in other studies, the high rates of HBsAg loss observed among patients with low pretreatment HBV DNA levels also open the door for potential novel treatment strategies aimed at this subgroup of patients. As spontaneous rates of HBsAg loss are limited, it is possible that initiating a finite course of NUC therapy may actually increase the chance of functional cure in this subgroup of patients. Whether such a strategy is worthwhile would require further study, preferably in a prospective (randomised) fashion, as there remains a risk of post-therapy relapse.

Finally, our findings are also important for the interpretation of studies conducted with novel antiviral compounds. As pretreatment viraemia appears to predict disease remission after therapy withdrawal, it will be of major importance to present the outcomes of future studies on finite therapy stratified by pretreatment HBV DNA levels to allow for comparisons across studies, cohorts, and compounds.

Although our study is relatively large and enrolled patients from centres in Europe and Asia, it does have several limitations. First, we limited our analysis to patients with HBeAg-negative CHB, and our findings therefore cannot be applied to patients with HBeAg-positive disease who achieved seroconversion during treatment. Furthermore, it is important to note that the patients with pretreatment HBV DNA <20,000 IU/ml enrolled in this study likely reflect a select subgroup of such patients with an indication for antiviral therapy, based on either ALT levels (73% had elevated ALT) or otherwise. In addition, HBV DNA levels may fluctuate over time, and patients with HBV DNA <20,000 IU/ml at the time of therapy initiation may have had higher levels during previous assessments. Of note, pretreatment ALT levels were not associated with off-treatment outcomes in the overall population or in the subset of patients with low pretreatment HBV DNA levels. Another potential limitation of this dataset is the relatively low number of non-Asian patients. Although we observed consistent results among Asian and Caucasian patients, with superior outcomes observed for patients with a baseline HBV DNA level <20,000 IU/ml, findings were non-significant in the relatively limited number of Caucasian patients, and the group of non-Asian/non-Caucasian patients was too small to analyse separately. Finally, the current cohort lacks detailed information on pretreatment stage of liver disease (information was missing in 309 cases), and we were therefore unable to analyse the association between stage of liver fibrosis and outcomes after therapy cessation.

In conclusion, our multicentre study shows that low pretreatment HBV DNA levels are associated with favourable outcomes after NUC cessation. Further studies are required to confirm these results in non-Asian populations. Our findings can be used to select patients for, and counsel patients about, their chances of successful finite NUC therapy.

## Financial support

The CREATE study was supported by Fujirebio. Materials for HBcrAg testing were provided free of charge to several participating centres. Fujirebio had no influence on CREATE study design, data collection, data analysis, writing of the current manuscript, or the decision to submit for publication.

## Authors’ contributions

Study design: MJS, SMC, CHC, BM, JYP, AK, WKS, YT, IC, MP, FvB, TB, FZ, SHA, GND, HW, MC, MFY, KA, AB, MB, TP, GP, BM, PC, MB, NSE. Collection of data: MJS, SMC, CHC, BM, JYP, AK, WKS, YT, IC, MP, FvB, TB, FZ, SHA, GND, HW, MC, MFY, KA, AB, MB, TP, GP, BM, PC, MB, NSE. Data analysis: MJS, SMC, CHC, BM, JYP, AK, WKS, YT, IC, MP, FvB, TB, FZ, SHA, GND, HW, MC, MFY, KA, AB, MB, TP, GP, PC, MB, NSE. Writing of the manuscript: MJS, SMC, CHC, BM. Critical review of the manuscript: JYP, AK, WKS, YT, IC, MP, FvB, TB, FZ, SHA, GND, HW, MC, MFY, KA, AB, MB, TP, GP, BM, PC, MB, NSE. Approval of the final version: all authors. Approval of the submission of the manuscript: all authors.

## Data availability statement

The data used for the current analysis were derived from previously published cohorts and clinical datasets. The data cannot be shared.

## Conflicts of interest

MJS has received speaker’s fees and research support from Roche, Gilead, BMS, and Fujirebio. SMC has nothing to disclose. JYP is an investigator in clinical trials sponsored by AbbVie, Gilead Sciences, Hanmi, and Norvatis. WKS has received speaker’s fees from Mylan and AstraZeneca; has provided consultancy for Abbott; has received speaker’s fees and provided consultancy for AbbVie; and has received speaker’s fees from, provided consultancy for, and received research funding from Gilead Sciences. YT reports lecture fees from Fujirebio, GlaxoSmithKline Pharmaceuticals Ltd, and Gilead Sciences, and research fees from Fujifilm Corp, Janssen Pharmaceutical K.K., Gilead Sciences, GlaxoSmithKline Pharmaceuticals Ltd, Sysmex, and Stanford Junior University. FvB has received research support from and provided consultancy for Roche. TB currently acts as an advisor to AbbVie, Alexion, Bayer, BMS, Gilead, Intercept, Janssen, MSD/Merck, Merz, Novartis, and Sequana Medical. He has received speaking honoraria from AbbVie, Alexion, Bayer, BMS, Eisai, Gilead, Intercept, Ipsen, Janssen, MSD/Merck, Merz, Novartis, Sirtex, and Sequana Medical in the past 2 years. He has received grant support from AbbVie, BMS, Gilead, Humedics, Intercept, Janssen, MSD/Merck, Merz, Novartis, and Sequana Medical. FZ is an advisor for Aicuris, Aligos, Antios, Assembly, Blue Jay, Evotec, Gilead, and GSK, and has received research grants from Assembly, Beam, Janssen, and Viravaxx. SHA has acted as an advisor and investigator for Gilead, Janssen, AbbVie, Roche, Assembly Biosciences, Arbutus, Brii, Vaccitech, GSK, Inovio, Aligos, Vir Biotechnology, SL Vaxigen, GeneOne Life Science, GreenCross, Yuhan, Samil, and Ildong. GND is an advisor or lecturer for Ipsen, Pfizer, Genkyotex, Novartis, and Sobi; has received research grants from AbbVie and Gilead; and has served as PI in studies for AbbVie, Novartis, Gilead, Novo Nordisk, Genkyotex, Regulus Therapeutics Inc, Tiziana Life Sciences, Bayer, Astellas, Pfizer, Amyndas Pharmaceuticals, CymaBay Therapeutics Inc, Sobi, and Intercept Pharmaceuticals. MB has received fees for consultancy/speakers bureau from AbbVie, Gilead, Janssen, EISAI-MSD, and Roche. HW has received research grants from Abbott, AbbVie, BMS, Gilead, Merck, Novartis, Roche, Roche Diagnostics, and Siemens; consultant fees from Abbott, AbbVie, BMS, Boehringer Ingelheim, Gilead, JJ/Janssen-Cilag, Merck/Schering-Plough, Novartis, Roche, Roche Diagnostics, Siemens, Transgene, and ViiV; and speaker fees from Abbott, AbbVie, BMS, Boehringer Ingelheim, Gilead, JJ/Janssen-Cilag, Merck/Schering-Plough, Novartis, Roche, Roche Diagnostics, Siemens, Transgene, and ViiV. MC has received personal fees from AbbVie, Bristol Myers Squibb, Gilead Sciences, Janssen-Cilag, Merck (MSD), Biogen, Falk Foundation, Boehringer Ingelheim, Siemens, and Spring Bank as well as grants and personal fees from Roche. MFY has provided consultancy for and/or received research funding from AbbVie, Arbutus Biopharma, Assembly Biosciences, Bristol Myers Squibb, Dicerna Pharmaceuticals, GlaxoSmithKline, Gilead Sciences, Janssen, Merck Sharp and Dohme, Clear B Therapeutics, and Spring Bank Pharmaceuticals, and has received research funding from Arrowhead Pharmaceuticals, Fujirebio Incorporation, and Sysmex Corporation. KA has received fees for consultancy/speakers bureau from Assembly, Aligos, Arbutus, Gilead, Immunocore, Janssen, Roche, Sobi, Spring Bank, and Vir, and research support from Abbott, Gilead, and MSD. AB has received research fees from Fujirebio, Gilead Sciences, and Janssen Pharma. MB reports consultancy and lecture honoraria from AbbVie, Arbutus, Gilead, Janssen, Merck/MSD, and Spring Bank. GP has served as an advisor/lecturer for AbbVie, Albireo, Dicerna, Gilead, GSK, Janssen, Ipsen, MSD, Novo Nordisk, Roche, and Takeda, and has received research grants from AbbVie and Gilead. CHC has nothing to disclose. BM has received speaker and/or consulting fees from Abbott Molecular, Astellas, Intercept, Falk, AbbVie, Norgine, Bristol Myers Squibb, Fujirebio, Janssen-Cilag, Merck (MSD), and Roche. He has also received research support from Abbott Molecular, Altona Diagnostics, Fujirebio, and Roche.

Please refer to the accompanying ICMJE disclosure forms for further details.
