# Supplementary material for: Lower pretreatment HBV DNA levels are associated with better off-treatment outcomes after nucleo(s)tide analogue withdrawal in patients with HBeAg-negative chronic hepatitis B: A multicentre cohort study
Source: JHEP Rep. 2023 May 12;5(8):100790. doi: 10.1016/j.jhepr.2023.100790 (PMC10362794; doi:10.1016/j.jhepr.2023.100790)
Supplement: Multimedia component 1 [file mmc1.pdf]

Supplementary materials

Lower pretreatment HBV DNA levels are associated with better off-treatment outcomes after nucleo(s)tide analogue withdrawal in patients with HBeAg-negative chronic hepatitis B: A multicentre cohort study

Milan J. Sonneveld[1*], Shao-Ming Chiu[2*], Jun Yong Park[3], Sylvia M. Brakenhoff[1], Apichat Kaewdech[4], Wai-Kay Seto[5], Yasuhito Tanaka[6], Ivana Carey[7], Margarita Papatheodoridi[8], Piero Colombatto[9], Florian van Bömmel[10], Thomas Berg[10], Fabien Zoulim[11], Sang Hoon Ahn[3], George N. Dalekos[12], Nicole S. Erler[13,14], Maurizia Brunetto[9], Heiner Wedemeyer[15], Markus Cornberg[15], Man-Fung Yuen[5], Kosh Agarwal[7], Andre Boonstra[1], Maria Buti[16], Teerha Piratvisuth[4], George Papatheodoridis[8], and Chien-Hung Chen[2*] and Benjamin Maasoumy[15*,]

for the CREATE study group

Table of contents

Fig. S1. Page 2
Table S1. Page 3
Table S2. Page 4
Table S3. Page 5
Table S4. Page 6

Fig. S1.

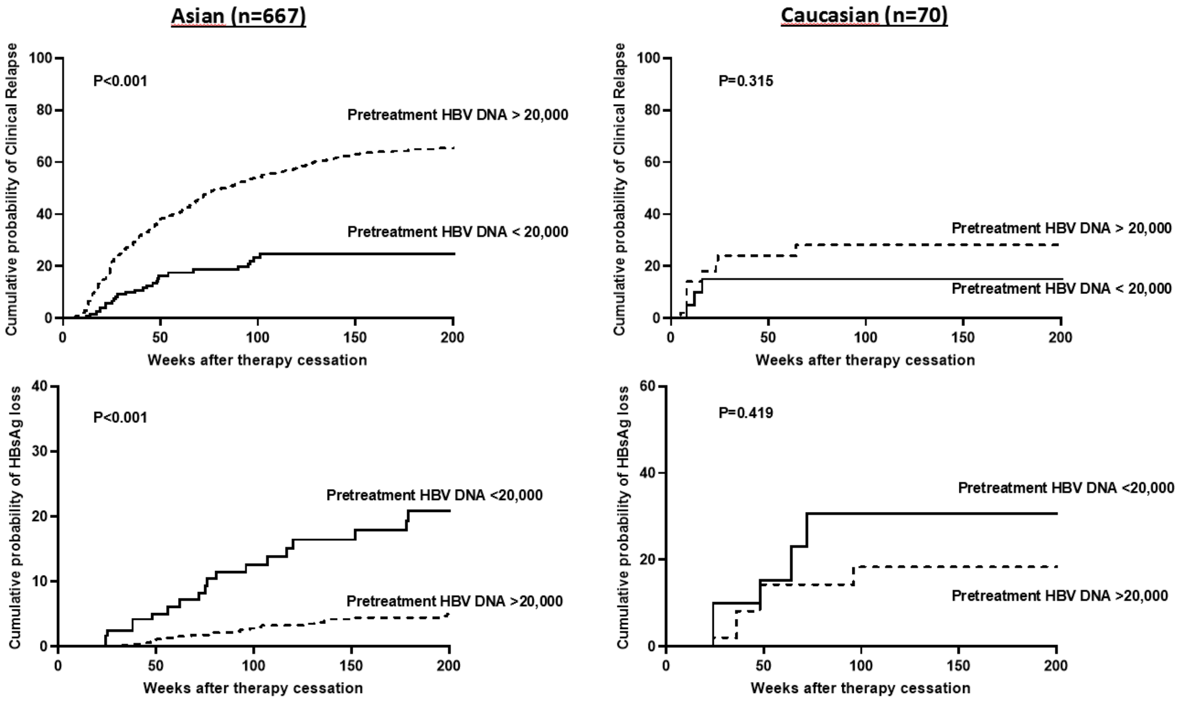

Fig. S1. Off-treatment outcomes according to pretreatment HBV DNA levels stratified by ethnicity. Cumulative probability of clinical relapse and HBsAg loss according to pretreatment HBV DNA levels for Asian (clinical relapse: p<0.001; HBsAg loss: p<0.001 by log-rank test) and Caucasian patients (clinical relapse: p=0.315; HBsAg loss: p=0.419 by log-rank test).

Table S1. Cohort characteristics by pretreatment HBV DNA level

Characteristics	Pretreatment HBV DNA		P
	<20,000 IU/mL (n=150)	>20,000 IU/mL (n=607)	
**Demography**			
Age (years), median (IQR)	53 (47 – 60)	53 (45 – 60)	0.685
Male	100 (67%)	465 (77%)	0.012
Duration of therapy (weeks, IQR)	166 (156 – 263)	158 (156 – 262)	0.802
Pretreatment ALT (median, IQR)	76 (26 – 138)	125 (83 – 260)	<0.001
Cirrhosis (n=448)	13 (15.7%)	10 (2.7%)	<0.001
**Ethnicity**			0.002
Asian	121 (81%)	546 (90%)	
Caucasian	20 (13%)	50 (8.2%)	
Other	9 (6.0%)	11 (1.8%)	
**Treatment**			0.244
ETV	77 (51%)	351 (58%)	
TDF	50 (33%)	188 (31%)	
other	23 (15%)	68 (11%)	
**HBV genotype (n=629)**			0.003
A	5 (4.0%)	5 (1.0%)	
B	69 (56%)	280 (55%)	
C	24 (19%)	160 (32%)	
D	18 (15%)	45 (8.9%)	
Other	8 (6.4%)	15 (3.0%)	
**End of treatment HBsAg**			0.001
<100 IU/mL	55 (37%)	145 (24%)	
>100 IU/mL	95 (63%)	462 (76%)	
**End of treatment HBcrAg**			<0.001
Undetectable	76 (51%)	161 (27%)	
Detectable	74 (49%)	446 (74%)	

Characteristics of the patients enrolled in this cohort. EOT, end of treatment. IQR, interquartile range. ETV, entecavir; TDF, tenofovir; HBV, hepatitis B virus. Comparisons performed using the Chi-squared test (for categorical variables) and the Mann-Whitney U test.

Table S2. Cohort characteristics by ethnicity

Characteristics	Ethnicity		P
	Asian (n=667)	Caucasian (n=70)	
**Demography**			
Age (years), median (IQR)	53 (45 – 59)	59 (49 – 65)	0.009
Male	507 (76.0%)	48 (68.6%)	0.170
Duration of therapy (weeks, IQR)	157 (156 – 209)	385 (267 – 512)	<0.001
Pretreatment ALT (median, IQR)	120 (81 – 246)	84 (43 – 164)	<0.001
**Treatment**			<0.001
ETV	402 (60.3%)	23 (32.9%)	
TDF	176 (26.4%)	45 (64.3%)	
other	89 (13.3%)	2 (2.9%)	
**HBV genotype**			<0.001
A	0 (0%)	3 (4.4%)	
B	348 (64.3%)	1 (1.5%)	
C	183 (33.8%)	1 (1.5%)	
D	1 (0.2%)	62 (91.2%)	
Other	9 (1.7%)	1 (1.5%)	
**End of treatment HBsAg**			0.799
<100 IU/mL	181 (27.1%)	18 (25.7%)	
>100 IU/mL	486 (72.9%)	52 (74.3%)	
**End of treatment HBcrAg**			<0.001
Undetectable	181 (27.1%)	44 (62.9%)	
Detectable	486 (72.9%)	26 (37.1%)	

Characteristics of the patients enrolled in this cohort. EOT, end of treatment. IQR, interquartile range. ETV, entecavir; TDF, tenofovir; HBV, hepatitis B virus. Comparisons performed using the Chi-squared test (for categorical variables) and the Mann-Whitney U test.

Table S3. Factors associated with clinical relapse

Variable	aHR (95% CI)	p
**Pretreatment HBV DNA < 20,000 IU/mL**	0.474 (0.320 – 0.704)	<0.001
**EOT HBcrAg undetectable**	0.615 (0.467 – 0.809)	<0.001
**EOT HBsAg < 100 IU/mL**	0.329 (0.227 – 0.476)	<0.001
**Age**	1.021 (1.010 – 1.033)	<0.001
**EOT ALT**	1.004 (1.000 – 1.008)	0.052
**TDF therapy**	1.375 (1.074 – 1.760)	0.011
**Gender**	1.115 (0.837 – 1.485)	0.458
**HBV genotype**		<0.001
A/D	Reference	
B	1.957 (1.154 – 3.317)	
C	1.134 (0.642 – 2.003)	

Factors associated with clinical relapse after nucleo(s)tide analogue withdrawal in multivariable Cox regression analysis. aHR: adjusted hazard ratio.

Table S4. Factors associated with HBsAg loss

Variable	aHR (95% CI)	p
**Pretreatment HBV DNA < 20,000 IU/mL**	2.169 (1.346 – 3.497)	0.001
**EOT HBcrAg undetectable**	1.977 (1.210 – 3.229)	0.006
**EOT HBsAg < 100 IU/mL**	9.811 (5.961 – 16.148)	<0.001
**Age**	0.983 (0.960 – 1.007)	0.172
**EOT ALT**	0.988 (0.974 – 1.003)	0.108
**TDF therapy**	0.773 (0.464 – 1.287)	0.322
**Gender**	1.319 (0.768 – 2.265)	0.316
**HBV genotype**		<0.001
A/D	Reference	
B	0.226 (0.116 – 0.441)	
C	0.379 (0.179 – 0.801)	

Factors associated with HBsAg loss after nucleo(s)tide analogue withdrawal in multivariable Cox regression analysis. aHR: adjusted hazard ratio.
